# Effect of Mineral Admixtures on the Mechanical and Shrinkage Performance of MgO Concrete

**DOI:** 10.3390/ma16093448

**Published:** 2023-04-28

**Authors:** Xuan Zhou, Zhongyang Mao, Penghui Luo, Min Deng

**Affiliations:** 1 College of Materials Science and Engineering, Nanjing Tech University, Nanjing 211800, China; 202061203292@njtech.edu.cn (X.Z.); mzy@njtech.edu.cn (Z.M.); 202162103025@njtech.edu.cn (P.L.); 2 State Key Laboratory of Materials-Oriented Chemical Engineering, Nanjing 211800, China

**Keywords:** mineral admixture, MEA, fly ash, mineral powder, self-shrinkage, compressive strength

## Abstract

Shrinkage deformation of concrete has been one of the difficulties in the process of concrete performance research. Cracking of concrete caused by self-shrinkage and temperature-drop shrinkage has become a common problem in the concrete world, and cracking leads to a decrease in the durability of concrete and even a safety hazard. Mineral admixtures, such as fly ash and mineral powder, are widely used to improve the temperature drop shrinkage of mass concrete; fly ash can reduce the temperature rise of concrete while also reducing the self-shrinkage of concrete, there are different results on the effect of mineral powder on the self-shrinkage of concrete, but the admixture of fly ash will reduce the strength of concrete, and mineral admixtures have an inhibitory effect on the shrinkage compensation effect of MgO expander(MEA). The paper investigates the effect of mineral admixtures on the mechanical and deformation properties of C50 mass concrete with a MgO expander(MEA), aiming to determine the proportion of C50 mass concrete with good anti-cracking properties under working conditions. The experiments investigated the effect of fly ash admixture, mineral powder admixture and MgO expander admixture on the compressive strength and deformation of concrete under simulated working conditions of variable temperature and analyzed the effect of hydration of magnesite in MgO expander and pore structure of cement paste on deformation. The following main conclusions were obtained: 1. When the concrete compounded with mineral admixture was cured under variable temperature conditions, the compounded 30% fly ash and mineral powder decreased by 4.3%, 6.0% and 8.4% at 7d age, and the compounded 40% fly ash and mineral powder decreased by 3.4%, 2.8% and 2.3% at 7d age, respectively. The incorporation of MEA reduced the early compressive strength of concrete; when the total amount of compounding remained unchanged, the early compressive strength of concrete was gradually smaller as the proportion of compounding decreased. 2. The results of concrete deformation showed that when the temperature rose, the concrete expanded rapidly, and when the temperature dropped, the concrete also showed a certain shrinkage, and the deformation of concrete basically reached stability at 18d. 3. The compounding of 30% fly ash and mineral powder As the compounding ratio decreases, the deformation of concrete increases, and the 28d deformation of concrete with a compounding ratio of 2:1 is 280 × 10^−6^, while the final stable deformation of concrete with a compounding ratio of 2:1 in compounding 40% fly ash and mineral powder is the largest, with a maximum value of 230 × 10^−6^, respectively. Overall, the concrete with a total compounding of 30% and a compounding ratio of 2:1 has the best shrinkage resistance performance.

## 1. Introduction

Concrete is one of the most commonly used construction materials in our life and has superior capacity under compressive loads, but it suffers from some serious defects. These defects are caused by drying shrinkage, thermal shrinkage, self-shrinkage and carbonation shrinkage, which cause the concrete volume to shrink [1,2]. This is the case with high-performance concrete (HPC), which has very good compatibility, mechanical properties and impermeability and is very widely used in engineering. However, the low water-cement ratio and additives make the hydration reaction much faster, which leads to a substantial and rapid temperature drop and a large self-shrinkage [3]. Self-shrinkage of high-performance concrete under certain conditions can lead to excessive concrete stresses, which can lead to cracks [4,5]. Early cracking seriously affects the durability and serviceability and even the safety performance of concrete structures. Therefore, expansion additives are used to compensate for shrinkage, traditionally with sulfate aluminate, aluminate clinker or grass-based [6]. These traditional expansion additives tend to be too dependent on wet curing, which occurs within 14 days of expansion, and early expansion is too fast; they do not compensate for the shrinkage of the concrete after 14 days of construction [7]. Among these expansion agents, MEA has the advantage of stable hydration products and long expansion time and is often used to prepare magnesium-based compensatory shrinkage cement, which is widely used in water conservancy construction [8]. MEA can improve concrete porosity and change concrete pore size distribution [9,10]. In the general mix, MEA content is 3–8%, which can not only compensate for the normal volume shrinkage but also improve the compressive performance, shrinkage performance and durability of concrete [11,12,13,14].

For large-volume concrete, with severe shrinkage deformation and high requirements for restriction, it is necessary to incorporate a large amount of MEA, but the delayed expansion of MEA can lead to shrinkage in concrete that is often overcompensated at a later stage, resulting in cracks and a reduction in all properties [15]. To prevent damage to concrete by MEA, some studies tend to reduce the delayed hydration expansion of MEA concrete but ignore the resistance to shrinkage that can enhance the concrete matrix and better coordinate the role of both, which should constrain expansion and compensate for significant shrinkage in the early stages of hydration [16,17]. The faster shrinkage of cement and slower expansion of MEA at ambient conditions can lead to shrinkage stress cracking, so we need to find an auxiliary cementitious material that coordinates the two. Fly ash and mineral powder, as a popular mineral admixture, are very likely to solve this problem depending on their properties in cement [18,19,20].

In recent years, the increasing cross-sectional size of the component, cement strength level and the amount of cement per unit volume resulted in a significant enhancement in the internal temperature rise during the hardening of concrete. This makes the difference between the standard curing temperature in the laboratory and the temperature in the actual structure, which also leads to a large gap between the strength of the specimen measured in the laboratory and the strength of the concrete in the actual structure. Based on this, this paper investigates the mechanical and shrinkage properties of mineral admixtures (fly ash and mineral powder) on MgO-based concrete in the context of the prevalence of concrete cracking in mass concrete and the actual curing temperature. The changes in expansion and mechanical properties under variable temperature conditions are studied macroscopically, and the effects of mineral admixtures on concrete are analyzed microscopically in terms of pore structure and micromorphology. By simulating the variable temperature environment inside the actual mass concrete, the concrete is mixed and formed, and then cured in the variable temperature environment; the properties of the concrete are measured, its development pattern is grasped, and its mechanism of action is studied, which can be used to guide the actual engineering projects in the future.

## 2. Materials and Methods

### 2.1. Raw Materials

The cement selected in this experiment is P·II52.5 Portland cement produced by Onoda of Nanjing, and the strength grade is C50; Fly ash is grade II fly ash of Nanjing Pukou power plant; The Minera powder is S95 grade Minera powder from the Pukou area of Nanjing; The expansion agent is MgO expansion agent produced by Jiangsu Subut New Materials Co. The fine aggregate is river sand with a fineness modulus of 2.7; the coarse aggregate is crushed stone with a continuous gradation of 5–31.5 mm; the water-reducing agent is polycarboxylic acid high-performance water-reducing agent produced by Jiangsu Subutech New Material Co. The chemical composition of raw materials is shown in Table 1.

### 2.2. Sample Preparation

In order to study the effect of mineral admixture on MgO-based concrete under variable temperature conditions, the total amount of cementitious material used is 450 kg/m^3^, and the amount of mineral admixture is 30% and 40% of the amount of cementitious material when fly ash is compounded with mineral powder, as this paper studies the effect of the admixture of mineral admixture on the performance of concrete, fly ash is extremely effective in improving the ease of ready-mixed concrete, but the concrete Early and medium-term strength development is slow, mineral powder relative to fly ash on the concrete strength development of each age faster, but poorer than fly ash, the two double admixture of concrete after each age strength and mix compatibility are better, the best ratio of 30% to 40% of the mass of cement [21]. In the compound admixture, the best mass ratio of fly ash to slag powder is 2:1, and the workability of C50 concrete is improved in the process of increasing the amount of external admixture from 0% to 30% [22]. So this paper also selects these two total admixture ratios, where the ratio of fly ash to mineral powder is 3:1, 2:1 or 1:1. MgO expander is mixed internally, and the admixture amount is selected as internal admixture, 8% of the cementitious material, which is higher than the conventional application. The sand rate was 0.39, the water-cement ratio was 0.32, and the water-reducing agent was blended at 2.8% of the cementitious material. The concrete mix ratios are shown in Table 2. The various raw materials were prepared and mixed in a mixer, and then water was added to continue the mixing. After the concrete was mixed, it was poured into a mold of a specific size and removed after one day.

### 2.3. Experimental Methods

#### 2.3.1. Heat of Hydration

The temperature difference generated by the heat of hydration released from the hydration of cementitious materials is the main cause of cracks in mass concrete, so a hydration calorimeter (TAM AIR, New Castle, DE, USA) was used to determine the early hydration process of concrete slurry containing MEA. Due to the small water-cement ratio of the cement slurry mixture, it could not be stirred in the calorimeter. Therefore, the calorimeter needs to be placed at a constant temperature of 20 °C for 24 h before testing, and 10.00 g of the test slurry is weighed with an accuracy of 0.01 g and loaded into the test cup of the reaction vessel to continuously follow the exothermic process of hydration of the net cement slurry over a period of time (72 h) to obtain a typical isothermal calorimetric curve, including the exothermic rate and the total exothermic amount with time.

#### 2.3.2. Mechanical Properties

According to Chinese standard GB/T 50081-2019, MgO-based concrete is used in Table 2 ratio; after concrete mixing, molding 150 mm × 150 mm × 150 mm cubic specimens, the molded concrete specimens together with touching tools are wrapped with cling film, input into a large plastic bag, tied with a tie, and put into the variable temperature curing box for curing. Through a whole temperature curve course, after which the specimens are removed and continued to be cured in 20 °C water. The WAW-600C microcomputer controlled electro-hydraulic servo universal testing machine (YiNuo, Jinan, China) was used to measure the 3d, 7d, 28d and 60d compressive strength of concrete. There were 3 concrete specimens in each group, and the compressive strength was taken as its flat value.

**Figure 1:**
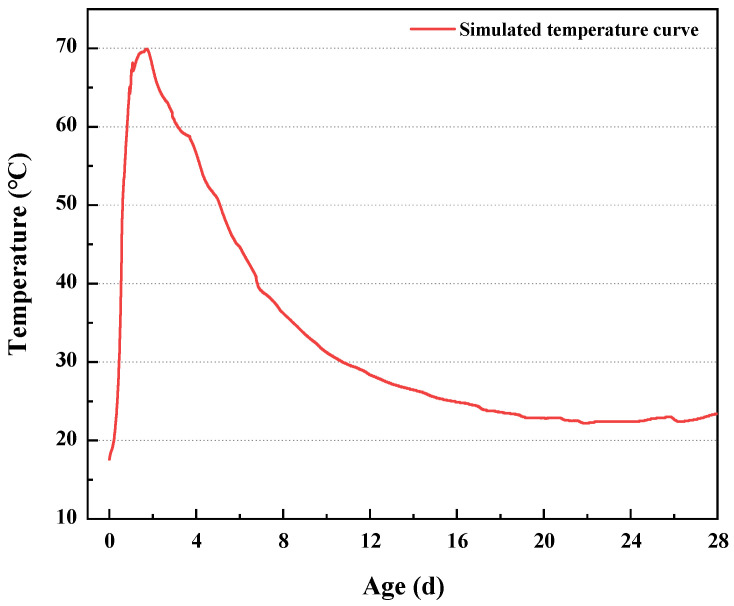
Variable temperature curve process.

Variable temperature curve course as shown in Figure 1, this curve comes from the actual large volume C5 concrete building internal temperature curve measured from the figure, 0–40 h time, the internal temperature of the concrete rose rapidly, reaching a maximum value in the 40thh, the highest temperature of 70 °C, after 40 h temperature began to decline until 18d, the internal temperature of the concrete down to a stable state, continue to measure until 28d, the temperature did not change significantly.

#### 2.3.3. Autogenous Deformation

**Figure 2:**
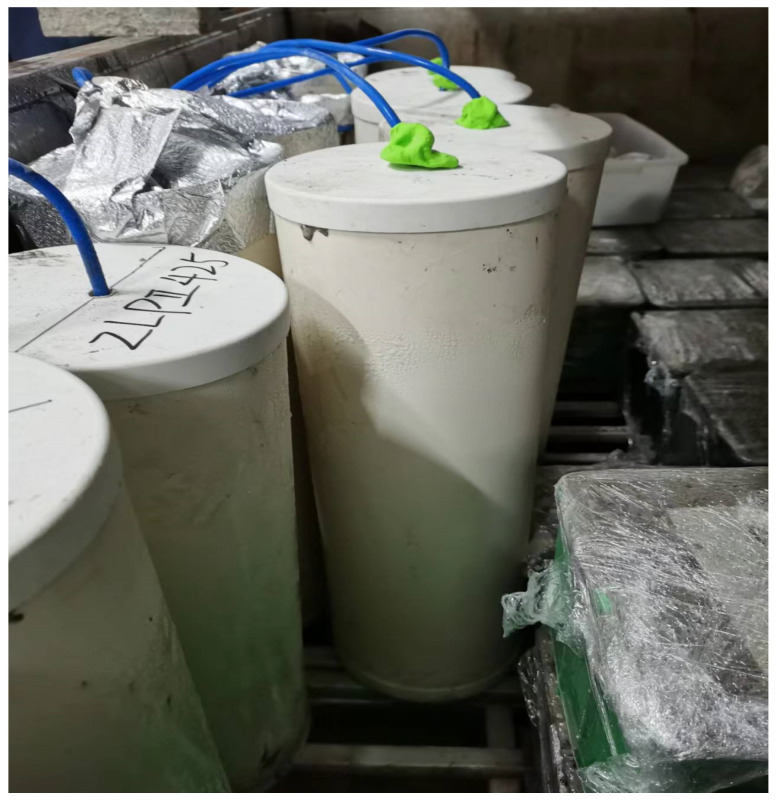
Concrete deformation sample.

Concrete was mixed and poured in Φ160 mm × 400 mm PVC pipe, pouring in the center of the specimen buried strain gage, and then a slight vibration until no air bubbles were generated. After the completion of the pouring, maintenance 20 °C environment, after the initial setting of the concrete will be closed with paraffin at both ends of the specimen to prevent the influence of water vapor, and then the specimen will go into the variable temperature curing box, continuous monitoring of the deformation of concrete, the concrete specimens are shown in Figure 2. After a whole temperature curve course, remove the specimen and continue curing at 20 °C environment until 28d.

#### 2.3.4. Thermal Analysis

The gravel-free concrete mixes were sealed up and placed in a variable temperature curing chamber using ages of 1d, 3d and 7d. The degree of hydration of MgO was evaluated by thermal analysis. The specimens were cut into slices, soaked in anhydrous ethanol for 3d and dried in a vacuum drying oven at 60 °C for 24 h. The specimens were ground into powder and sieved through a 0.08 μm square hole sieve. The DSC-TG method was used to heat the specimens under the N_2_ atmosphere with a temperature rise rate of 20 °C/min from 30 °C to 1000 °C.

Since the temperature in the natural environment shows periodic changes, the hydration of MEA will lead to constant changes in the volume of concrete. In order to be able to continuously track the volume deformation of MgO concrete, this experiment uses the MCU-32 automatic measuring instrument produced by Nanjing GeNan Industrial Company in China. To track the strain gauge, strain variables of the test and the acquisition time interval is 1 h. Since the temperature change under natural conditions will cause the volume of concrete to deform, the result calculated by Equation (1) is the strain variation of the concrete specimen after excluding the temperature deformation.


(1) $$\varepsilon_{ml}=k\times ∆F+(b-\alpha )∆T$$

In Equation (1) $\varepsilon_{ml}$ —Strain of concrete after deducting the effect of temperature.

k —Measurement sensitivity of strain gauges (10^−6^/F).

∆F —Measurement difference of strain gauges.

b —Thermal expansion coefficient of strain gauge (13.5 × 10^−6^/°C).

α —Coefficient of thermal expansion of the concrete specimen under test (5.5 × 10^−6^/°C).

#### 2.3.5. Pore Structure

Determination of pore structure and porosity of concrete mixes by Poremaster GT-60 mercury compression meter (Quantachrome, Boynton Beac, FL, USA). The samples of 1d age were cut into small pieces of 2–3 mm in size, put into anhydrous ethanol to terminate hydration, soaked for 3d, and then dried in a vacuum drying oven at 60 °C for 24 h before being taken out for testing. The pore pressure was 415 Mpa, and the pore range was 0.007 μm–360 μm.

#### 2.3.6. SEM Morphology

Samples were taken from the pressed concrete specimens, soaked in anhydrous ethanol to terminate hydration, soaked for 3d, dried at a temperature of 60 °C for 24 h and then removed, the specimens were coated with gold, and the microscopic morphology of concrete was observed using a JSM6480 scanning electron microscope(Thermo Fisher Scientific, Waltham, MA, USA), and the relationship between strength and concrete structural densities was desired.

## 3. Results and Discussion

### 3.1. Hydration Heat

**Figure 3:**
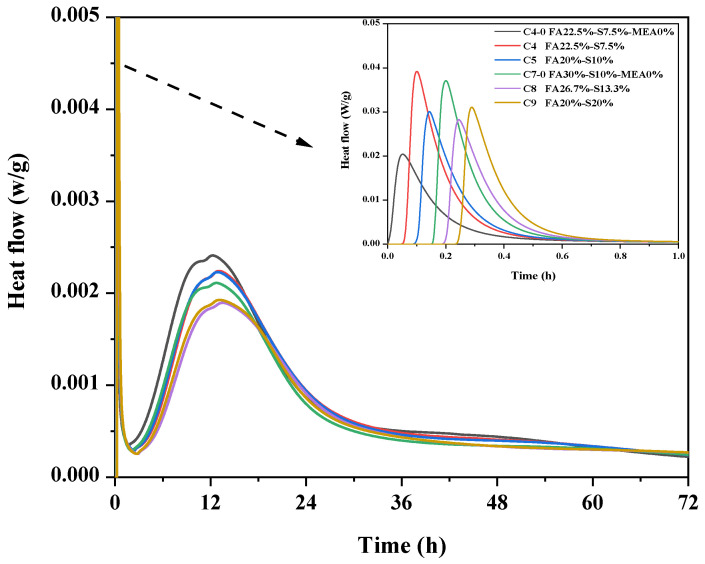
Effect of mineral admixtures on heat flow rate.

Figure 3 shows the heat flow in 3 d for different mineral admixtures. The results show that the induction period occurs mainly within 2 h. The accelerated period has a faster exothermic rate and lasts for about 11 h. The accelerated and delayed periods take a total of 13 h, which is basically consistent with the initial setting time of concrete. The longer induction time is due to the fact that less free water in the system inhibits the diffusion of Ca^2+^ and OH^−^. On the other hand, the high concentration of SP also inhibited the nucleation growth of unhydrated cement particles and the formation of Ca(OH)_2_, which led to a slower hydration process in the HPC system [23]. It is presumed that the early expansion of concrete may be caused by the temperature increase; after that, as the hydration of the system enters the deceleration period, which lasts about 20 h, at the early stage of deceleration, the exothermic heat of hydration is greater than the exothermic heat, so the internal temperature of concrete is still rising until the maximum temperature, and when the exothermic heat is smaller than the exothermic heat, the temperature starts to fall until the room temperature. It can be presumed that concrete accelerates expansion in the early stage of hydration and starts shrinking in the late stage of hydration. Eventually, the whole system of cementitious material hydration is basically stable, and the heat dissipation is small.

As shown in Figure 3, C4 leads to a slowing down of the heat flow of hydration when 8% MEA is incorporated compared to C4-0. Additionally, increasing the total amount of mineral admixture to 40% did not show a longer induction period and acceleration cycle but only a significant reduction in the hydration heat flow. As seen in the figure, changing the admixture of fly ash and mineral powder thus leads to a change in the release of hydration heat flow; when the admixture of fly ash is 20% and the admixture of mineral powder is 20%, the heat release peak is significantly wider and shows longer induction period and acceleration period. For MgO-based shrinkage-compensated cement, the incorporation of mineral admixture enhances the aluminate reaction and diminishes the effect of MEA in accelerating the thermodynamic reaction [24]. Therefore, changing the ratio of mineral admixtures can reduce the possibility of MgO-based large-volume concrete cracking due to temperature stresses.

### 3.2. Autogenous Shrinkage

**Figure 4:**
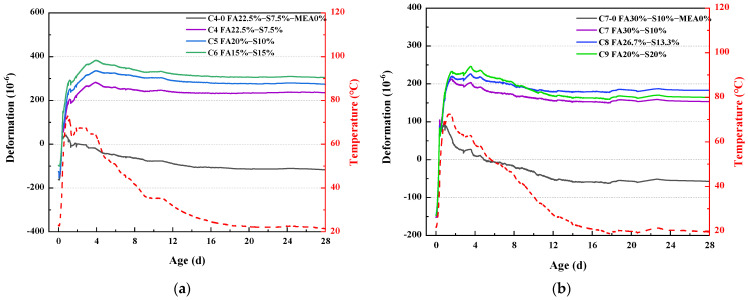
Autogenous Shrinkage of fly ash and S95 ore pulverized concrete after deducting the influence of temperature. (**a**) Total dosage is 30%; (**b**) Total dosage is 40%.

Figure 4 depicts autogenous shrinkage of fly ash and S95 ore pulverized concrete after deducting the influence of temperature. Figure 4a depicts the self-deformation of the concrete compounded with 30% fly ash and S95 mineral powder in 28d net of temperature effects. The red line in the figure shows the temperature change curve for the whole test, which shows that the test reaches the highest temperature at 1d, about 75 °C, and then proceeds to a slight decrease to room temperature of 20 °C at 18d. All samples were expanding rapidly during the early temperature rise period, but the C4-0 sample showed a lack of subsequent expansion, which was smaller than that of the MEA-doped sample, and eventually began to shrink after experiencing the highest temperature and showed contraction after 28d. The early expansion of the C6 sample was the largest, indicating that fly ash had an inhibitory effect on early expansion under variable temperature conditions. The samples doped with MEA eventually exhibited expansion after 28d, which indicates that MEA compensates well for early shrinkage.

Figure 4b depicts the self-deformation of the concrete compounded with 40% fly ash and S95 mineral powder in 28 days net of temperature effects. The C9 samples started with greater volume expansion than C8 and C7, and subsequently, the generated Ca(OH)_2_ and Mg(OH)_2_ stimulated the volcanic ash activity of the fly ash, producing large amounts of C-S-H, C-A-H, and M-S -H [25,26]. The maximum volume deformation of concrete with 30% secondary fly ash and 10% mineral powder without MEA is 90 × 10^−6^, and the stable value is −50 × 10^−6^, showing a shrinkage state. In the proportion of concrete mixed with MEA, the maximum volume deformation of concrete mixed with 30% second-grade fly ash and 10% mineral powder is 220 × 10^−6^, and the stable value is 150 × 10^−6^. The maximum volume deformation of concrete with 26.7% second-grade fly ash and 13.3% mineral powder is 230 × 10^−6^, and the stable value is 180 × 10^−6^. The maximum volume deformation of concrete with 20% secondary grade fly ash and 20% mineral powder is 250 × 10^−6^, and the stable value is 170 × 10^−6^. It can be seen from the above data that the concrete mixed with 26.7 secondary fly ash and 13.3% mineral powder has the smallest shrinkage with the decrease in temperature, and the concrete has the best shrinkage resistance.

### 3.3. Compressive Strength

**Figure 5:**
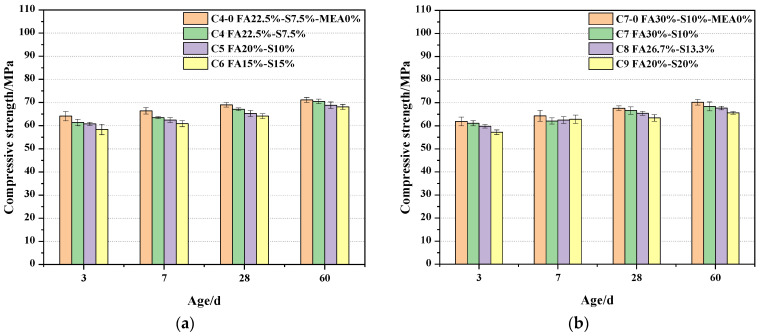
Compressive strength of fly ash and S95 ore pulverized concrete. (**a**) Total dosage is 30%; (**b**) Total dosage is 40%.

Figure 5a depicts the compressive strengths at 3d, 7d, 28d and 60d for concrete compounded with 30% fly ash and S95 mineral powder. The compressive strength gradually decreases with increasing the ratio of fly ash to mineral powder 3:1, 2:1 and 1:1, and the incorporation of MEA also leads to a decrease in compressive strength. For the mineral admixture concrete system, the compressive strength of C4, C5 and C6 samples at 3d age decreased by 2.76 Mpa, 3.3 Mpa and 5.6 Mpa, respectively, compared to C4-0 samples, while the compressive strength of C4, C5 and C6 samples at 60d age decreased by 0.6 Mpa, 2.3 Mpa and 3 Mpa, respectively, compared to C4-0 samples. The effect was greater at the early stage and had almost No effect. It was found that the adverse effect on the compressive strength of concrete may be due to the fact that MEA reduces the volume fraction of cement, while hydration products, such as magnesium hydroxide, contribute less to the strength [27]. In addition, it is also possible that the hydration products have relatively small crystals and are less strong than the cement hydration products, resulting in lower mechanical properties [28].

Figure 5b shows the histogram of compressive strength with age for concrete with 40% total mineral admixture under variable temperature conditions. We found that the compressive strength of concrete mixed with MEA was generally higher than that of the unmixed 40% mineral admixture, but unlike the 30% mineral admixture, the compressive strength of concrete at age 7d surprisingly increased with the decrease of fly ash admixture and the increase of mineral powder admixture. The compressive strength at the 3d age of concrete mixed with MEA decreased by 1.3%, 3.4% and 7.6%, respectively, compared with that of unmixed. The compressive strength of 3d concrete decreased with decreasing amount of secondary fly ash and an increasing amount of mineral powder. 3d compressive strength of concrete mixed with 30% secondary fly ash and 10% mineral powder was the highest in concrete mixed with MEA. 28d age, for the blank group, they decreased by 1.5%, 3.3% and 6.2%, respectively. At the age of 60d, the compressive strength of the concrete mixed with MEA was lower than that of the blank group by 2.6%, 3.6% and 6.6%, respectively. It indicates that the massive admixture of fly ash has a positive effect on the development of concrete strength under variable temperature conditions.

### 3.4. XRD

**Figure 6:**
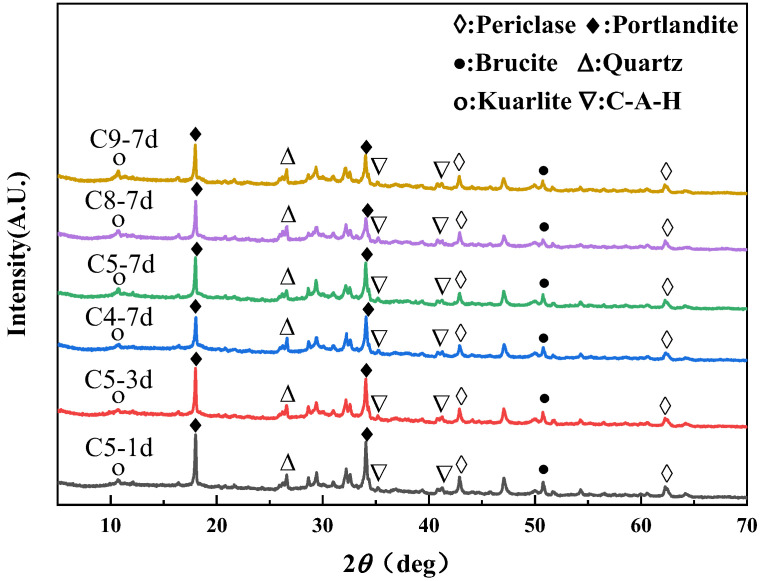
XRD spectra of C5, C4, C8 and C9.

Figure 6 describes the XRD patterns of C5, C4, C8 and C9 samples at 1d, 3d and 7d. The unhydrated magnesite in C4 samples exceeded C5 on day 7. With the increase of fly ash content, the unhydrated magnesite content in cement slurry increased, indicating that fly ash inhibited the hydration of MEA at the early stage of the variable temperature environment. The unhydrated cubic magnesite in the C8 sample exceeded C5 on the 7th day, which was also the concrete with a 2:1 dosage ratio. The content of unhydrated cubic magnesite in the cement slurry with a total content of 30% was lower than that in the cement slurry with a total content of 40%, indicating that mineral admixtures could inhibit the hydration of MEA. In the MgO-SiO_2_-H_2_O system, MgO can not only be chemically synthesized into Mg(OH)_2_ but also react with SiO_2_ to form M-S-H. Among them, the energy required to complete the second reaction is less than the first one [29]. With the addition of fly ash, the CaO content decreases and the SiO_2_ content increases. This way, the residual SiO_2_ combines with MgO and Mg(OH)_2_ as much as possible to form M-S-H [30,31,32,33,34].

### 3.5. Hydration Degree of MgO

**Figure 7:**
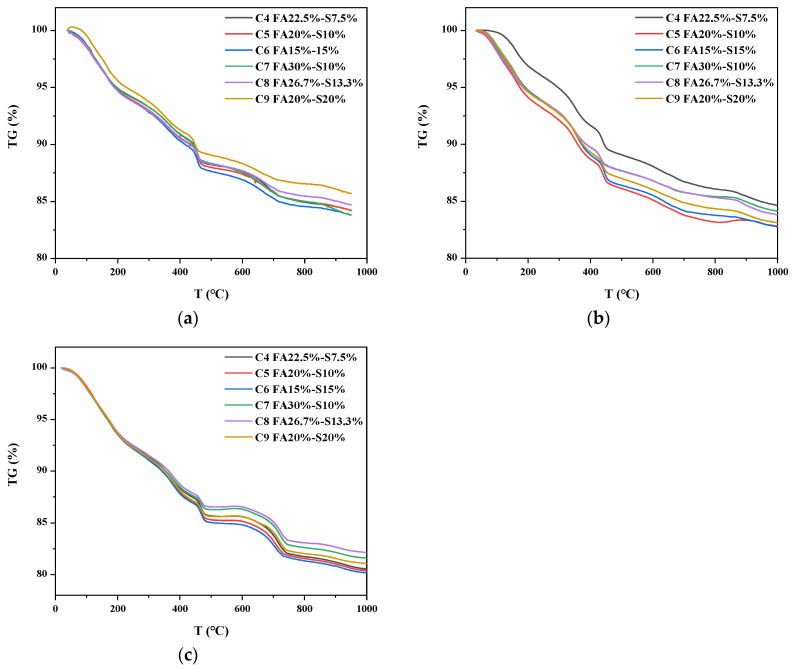
Hydration product contents at different ages. (**a**) 1 day of age; (**b**) 3 days of age; (**c**) 7 days of age.

The TG curves of C4, C5, C6, C7, C8 and C9 samples at d 1d, 3d and 7d are shown in Figure 7. In the temperature range of 30–330 °C, some hydration products, for example, C-A-H, C-S-H and Afm, decompose when heated to a certain temperature [35]. What we need to understand is that Mg(OH)_2_ and Ca(OH)_2_ undergo thermal decomposition at a temperature range of 330–420 °C and 420–500 °C, respectively [36,37].

**Figure 8:**
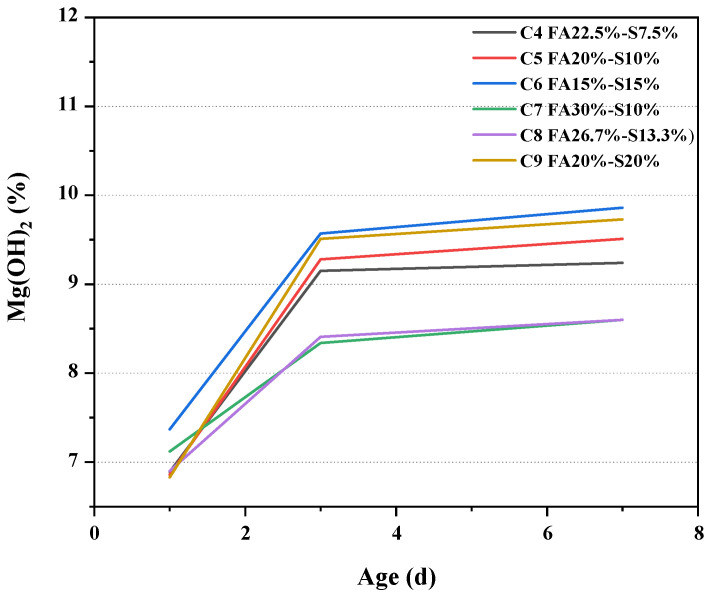
The content of Mg(OH)_2_ at different ages.

The equations of the calculation equation are displayed in Equations (2) and (3). The fraction of the mass of the sample at 1d age under variable temperature conditions is displayed in Table 3. Therefore, the content of Mg(OH)_2_ can be calculated according to Equation (4) and Table 3, and the results are shown in Figure 8.

(2) $${Mg(OH)}_{2}\to MgO+H_{2}O$$

(3) $${Ca(OH)}_{2}\to CaO+H_{2}O$$

(4) $$W_{{Mg(OH)}_{2}}={∆W}_{330–420{}^{\circ}C}\frac{M_{{Mg(OH)}_{2}}}{M_{H_{2}O}}$$

In Equations (2)–(4) $W_{{Mg(OH)}_{2}}$ is the mass fraction of Mg(OH)_2_ (%); $M_{{Mg(OH)}_{2}}$ and $M_{H_{2}O}$ are the molar masses of Mg(OH)_2_, H_2_O (g/mol). ${∆W}_{330–420{}^{\circ}C}$ expresses the mass loss fraction in the temperature range of 330–420 °C.

Figure 8 shows the content of Mg(OH)_2_ in the pure pulp of Grade II fly ash and mineral powder cement mixed with 30% and 40% at variable temperatures. It can be seen from the figure that, The Mg(OH)_2_ content of concrete slurry with 22.5% secondary fly ash, 7.5% ore powder, 20% secondary fly ash 10% ore powder, 15% secondary fly ash, 15% ore powder, 30% secondary fly ash 10% ore powder, 26.7% secondary fly ash 13.3% ore powder and 20% secondary fly ash 20% ore powder at 7 days age, respectively is 9.24%, 9.51%, 9.86%, 8.60%, 8.60% and 9.73%. 15% secondary fly ash has the largest Mg(OH)_2_ content and corresponding deformation, with an expansion of 350 με at 7d, while 30% secondary fly ash 10% mineral powder has the lowest Mg(OH)_2_ content and the lowest deformation, with an expansion of 170 με at 7d. The curve in the figure also decreases with the increase of secondary fly ash content and the decrease of mineral powder content. For example, the C6 curve in the figure is above C5. It can be seen that the incorporation of fly ash weakens the hydration process of magnesium oxide to some extent. Although the Mg(OH)_2_ content of 15% secondary fly ash and 20% secondary fly ash is very similar at 3d age, the expansion of the former is 120 με larger than that of the latter. From the point of view of total shrinkage compensation, the concrete mixed with 20% secondary fly ash and 10% mineral powder is the most suitable.

### 3.6. Microstructure

#### 3.6.1. Pore Structure

**Figure 9:**
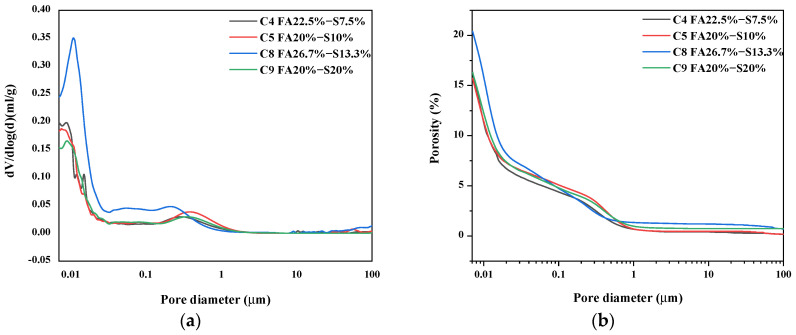
Pore size structure of slurries with different mineral admixtures. (**a**) Total dosage is 30%; (**b**) Total dosage is 40%.

Figure 9a depicts the pore structure of the different mineral admixtures at the age of 7d. It can be seen that the pore sizes of the samples are mainly distributed in the range of 0.007 to 0.03μm. Compared with the C5 sample, the pore size peak of the C9 sample is significantly shifted to the right, indicating that the multiple admixtures of mineral powder lead to larger pores of the whole system. During the admixture of fly ash, the pores become less, which will make the concrete denser and strengthen the mechanical properties of concrete. It indicates that fly ash can stabilize the MEA and improve structural compactness. As in Figure 9b, the magnitude of porosity in the samples is C4, C5 < C9, indicating that the increase in the total admixture of mineral admixture leads to an increase in the porosity of cement paste in concrete but a significant decrease in the average pore size. Increasing the admixture of mineral powder also leads to an increase in porosity, which is not conducive to the stability of the concrete structure. Overall, the distribution curve of pore size is generally shifted towards smaller pore sizes. Therefore, a larger proportion of fly ash can mitigate the self-shrinkage of concrete more effectively.

#### 3.6.2. SEM

**Figure 10:**
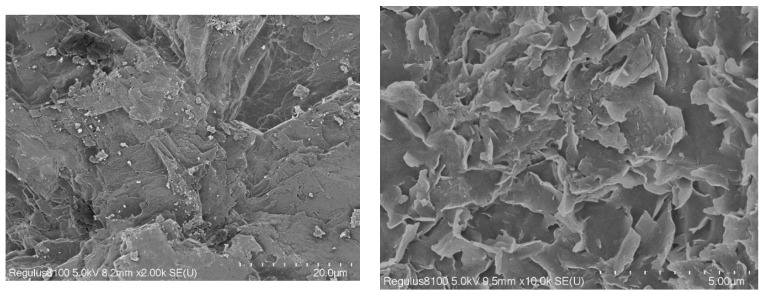
SEM images of test specimens.

In order to have a deeper understanding of the differences in the mechanical strength development of C4 and C6 samples from 3d to 60d, Figure 10 shows the SEM observation. There is no flocculation on the surface of C4 samples at 60 days, and there is no fracture layer at the links, which indicates that the multiple admixtures of fly ash have a positive effect on the later tissue growth of C5 samples. While SEM of the C6 sample at 60 days, flocs were formed on the surface, and these flocs were flake structures, and this structure was not conducive to the development of concrete strength. The loose structure of these Mg(OH)_2_ crystals is related to the hydration of MgO, which leads to premature swelling. Strongly demonstrates the positive effect of fly ash on improving the structural compactness of concrete. There are two possible reasons for this positive effect. One is that fly ash attenuates the delayed over-swelling by reducing Mg(OH)_2_, and the other is that it forms a better cement matrix.

## 4. Conclusions

In this topic, the effects of mineral admixtures on MgO concrete under variable temperature conditions were systematically studied from some aspects, such as shrinkage properties, mechanical properties and microstructure. Based on the experimental results, the following conclusions can be obtained.

1. Compared with the sample without MEA, the addition of MEA can significantly reduce the heat flow of cement, increase the mineral admixture can, reduce the heat of hydration and inhibit the delayed overexpansion of concrete, improve the later mechanical properties of concrete, and reduce the risk of cracking of concrete.
2. The results of the self-shrinkage show that concrete expands sharply and then shrinks under variable temperature conditions. However, concrete without MEA eventually exhibits shrinkage, while concrete with MEA exhibits expansion. The incorporation of mineral admixtures prevents cracking of the concrete due to self-shrinkage and prevents delayed over-expansion of the concrete in the later stages.
3. Under variable temperature conditions, the addition of mineral admixtures will lead to a decrease in the mechanical properties of concrete, but the amount of reduction is very small and meets the actual needs. Under variable temperature conditions, the mechanical properties decrease with the decrease of fly ash content, indicating that the incorporation of fly ash is conducive to the mechanical properties of concrete.
4. The XRD and TG results show that under variable temperature conditions, fly ash leads to weaker shrinkage resistance of concrete in the early stages by reducing the hydration of MEA inside the concrete, and in the later stages, mineral admixtures form stronger concrete cementing substances through secondary reactions. Overall, fly ash can harmonize the relationship between shrinkage resistance and shrinkage of concrete.
5. MIP analysis shows that the porosity of the C5 sample with 10% ore powder of 20% fly ash is 16%, while the porosity of 20% ore powder of 20% fly ash is 17%. Increasing the ore powder content can increase the porosity, indicating that a large amount of ore powder is not conducive to the thinning of pore size. The addition of fly ash makes the pore size refined continuously, the porosity reduced, and the hydration products closer to each other. It will make the structure of concrete more dense and strengthen the mechanical properties of the content.

## 5. Recommendation

By studying the laws of mineral admixtures on the mechanical and shrinkage properties of concrete under variable temperature conditions. When working on mass concrete, we need to fully consider its internal temperature rise due to the hydration of cement, where temperature gradient changes can lead to cracking of the concrete, thus, affecting the durability performance of concrete and even safety hazards. In order to reduce the temperature rise of concrete, we can take the internal buried cooling pipes and also add mineral admixtures. The above study found that adding 30% of mineral admixture is the most suitable, and 30% of the total amount of compounding, compounding ratio 2:1, and the best case, its final deformation for the expansion state, to solve the cracking of concrete because of shrinkage, also will not occur excessive expansion of concrete, its mechanical properties are also to meet the project requirements.

## Figures and Tables

**Table 1:** Chemical compositions of raw materials.

Material	SiO_2_ (%)	Al_2_O_3_ (%)	Fe_2_O_3_ (%)	CaO (%)	MgO (%)	K_2_O (%)	Na_2_O (%)	SO_3_ (%)	Loss (%)
Cement	18.55	3.95	3.41	65.32	1.01	0.72	0.18	2.78	2.88
Fly ash	44.06	42.06	2.91	3.80	0.40	0.49	0.16	0.75	2.48
Mineral powder	33.39	11.89	0.63	41.51	8.82	0.53	0.67	/	0.28
MEA	3.87	1.03	0.88	1.98	89.37	0.08	/	0.06	2.38

**Table 2:** Mix proportions of concrete.

Name	Cement (kg/m^3^)	Fly Ash (kg/m^3^)	Mineral Powder (kg/m^3^)	MEA (kg/m^3^)	Water (kg/m^3^)	Water Reducer
C4-0	315	101.2	33.8	0	144	2.8%
C4	279	101.2	33.8	36	144	2.8%
C5	279	90	45	36	144	2.8%
C6	279	67.5	67.5	36	144	2.8%
C7-0	270	135	45	0	144	2.8%
C7	234	135	45	36	144	2.8%
C8	234	120	120	36	144	2.8%
C9	234	90	90	36	144	2.8%

**Table 3:** Mass fraction of 1-day-old samples at different temperatures.

Name	C4	C5	C6	C7	C8	C9
330 °C	92.67%	92.36%	92.20%	92.67%	92.32%	93.07%
420 °C	90.53%	90.23%	89.91%	90.46%	93.18%	90.95%

## Data Availability

The data presented in this study are available on request from the corresponding author.
